# Axial Tracheids Widening Across Vein Orders in *Ginkgo biloba* Leaves and Their Relationship with Hydraulic Path Length

**DOI:** 10.3390/biology15080598

**Published:** 2026-04-10

**Authors:** Gusang Qunzong, Yuchen Cao, Qianhong Guo, Mengying Zhong

**Affiliations:** Key Laboratory of Biodiversity and Environment on the Qinghai-Tibetan Plateau, Ministry of Education, School of Ecology and Environment, Xizang University, Lhasa 850000, China; gusang@utibet.edu.cn (G.Q.); 13228918716@163.com (Q.G.)

**Keywords:** xylem conduit, tracheid hydraulic diameter, axial widening, hydraulic path length, vein order, allometric relationship

## Abstract

Leaves of vascular plants exhibit a tip-to-base xylem conduit widening pattern, which minimizes hydraulic resistance as leaves expand. The extent to which different leaf vein orders conform to this pattern remains largely unexplored, particularly in species with dichotomously branching veins. In this study, we focused on *Ginkgo biloba*, a model species with dichotomous venation, to explore tracheid variation across leaf vein orders and its linkage with hydraulic path length. Across 108 leaves from three sites, tracheid hydraulic diameter (*D*) scaled significantly with hydraulic path length (*L*) at an overall scaling slope of 0.33 (95% CI: 0.31–0.34, *r*^2^ = 0.53, *p* < 0.001). Scaling exponents differed significantly among sites and vein orders, and *D* decreased progressively with increasing vein order. This study provides a potentially valuable approach for investigating the effects of hydraulic path length on xylem conduit diameter and offers a framework for subsequent research on plant venation and hydraulic transport networks.

## Simple Summary

Many plants possess a specialized structure-the xylem conduit-in their leaves that facilitates efficient water transport from the leaf base to the tip. However, our understanding of how this structure functions in plants with unusual leaf vein patterns remains limited. To fill this knowledge gap, we focused on Ginkgo (*Ginkgo biloba*), an ancient relict plant species, to investigate how water-conducting structures vary from the leaf base to the tip. We collected and measured 108 ginkgo leaves from three distinct sites. Our observations revealed that the water-conducting tracheids are wider near the leaf base and narrower in higher-order veins, and tracheid diameter scales positively with hydraulic path length. Specifically, as the vein order increases, tracheids become progressively narrower from the main vein at the leaf base to the tiny veins at the leaf edge. Our findings improve our understanding of the structural adaptations that ensure efficient water transport in leaves, providing new insights into this fundamental physiological process in plants.

## 1. Introduction

Efficient and safe water transport is a fundamental prerequisite for sustaining the normal growth, development, and physiological activities of vascular plants. As the primary structural units responsible for water and nutrient transport, xylem conduits (including vessels and tracheids) play a pivotal role in determining hydraulic efficiency and plant adaptability to diverse environments. To minimize transport resistance and maximize transport efficiency, the construction of xylem conduits in vascular plants tends to maximize the exchange surface area for water and nutrients while reducing transport distance [1,2,3,4,5]. This pattern ensures that water can flow smoothly from the leaf base to the tip, supporting efficient photosynthesis and overall plant performance.

Under long-term natural selection, plant xylem conduits have evolved a structural characteristic of axial widening from the apex to the base, from leaves to stems, and further to roots. This widening pattern is independent of species and habitat [1,5,6,7,8]. The relationship between tracheid conduit diameter (*D*) and distance from the tip (*L*) generally follows a power-law function:

*D* ∝ a*L*^b^,

where a is the normalization constant and b represents the scaling exponent [1,9]. The functional significance of this axial widening lies in the fact that hydraulic conductance is highly sensitive to conduit dimensions; according to the Hagen-Poiseuille law, the hydraulic conductance of a single conduit is proportional to the fourth power of its diameter [10,11]. Therefore, even minor increases in tracheid diameter towards the leaf base can disproportionately reduce total hydraulic resistance and optimize water transport efficiency. Although conduit diameter and plant size show high plasticity across species and individuals, this axial widening pattern is highly convergent [4,9,12]. Specifically, the power-law relationship between basal conduit diameter and plant size is more consistent than that of environmental conditions or diverse life strategies [4,13,14]. Additionally, the rate of conduit widening is not uniform along the axial direction: it is highest near the leaf tip and gradually decelerates toward the base, a feature that further optimizes hydraulic performance [13,15,16]. However, it remains unclear whether the tip-to-base conduit widening pattern, widely documented in the complex reticulate venation of angiosperms, consistently governs the water-transport architecture across different vein orders in gymnosperms with primitive, non-anastomosing dichotomous venation. Here, we hypothesize that *Ginkgo biloba* leaves maintain a highly organized hydraulic hierarchy where tracheid diameter scales positively with hydraulic path length across all vein orders to minimize total transport resistance.

Despite substantial progress in understanding the tip-to-base xylem conduit widening pattern, significant research gaps remain. Most existing studies have focused on angiosperms with reticulated or pinnate venation systems [3,15,17]. Relatively few investigations have been conducted on gymnosperms, especially species with dichotomous venation—a primitive venation type that differs markedly from the reticulated venation of most angiosperms. Dichotomous venation is characterized by repeated branching into two equal parts, which may affect the distribution and widening pattern of tracheids across different vein orders. Therefore, whether the tip-to-base widening pattern holds consistently across all vein orders in dichotomously veined leaves remains largely unexplored. Furthermore, previous studies have rarely quantified the allometric relationship between tracheid diameter and hydraulic path length across different vein orders, nor have they systematically compared intraspecific variation in this pattern among different habitats or between individual plants. This lack of research limits our understanding of the generality and adaptability of the tip-to-base widening pattern in gymnosperms.

As a primitive gymnosperm and a renowned “living fossil”, *Ginkgo biloba* retains ancestral morphological traits that make it a premier model for investigating the evolutionary conservation of xylem hydraulic architecture. Unlike the complex, reticulate venation in angiosperms (e.g., *Populus* species) or the simplified parallel venation in conifers (e.g., *Pinus* species) networks, *Ginkgo biloba* exhibits a regular, open dichotomous venation. This architecture is defined by successive equal branching of veins that extend toward the lamina margin without forming secondary anastomoses. Such a configuration creates a structurally transparent and highly organized hydraulic hierarchy [18], facilitating the unambiguous identification of vein orders and providing an ideal framework to test the universality of the tip-to-base conduit widening pattern within primitive venation architectures.

In this study, we analyzed 108 leaves from 18 *Ginkgo biloba* individuals across three climatically distinct sites (Beijing, Lanzhou, and Lhasa) to characterize the hydraulic architecture of its dichotomous venation. Our primary objective was to quantify the allometric scaling relationship between tracheid hydraulic diameter (*D*) and hydraulic path length (*L*) across all vein orders, specifically testing two core predictions: (1) tracheid diameter exhibits a consistent tip-to-base axial widening pattern, with *D* scaling positively with *L*; (2) tracheid diameter decreases progressively with increasing vein order, aligning with the hierarchical organization of the leaf’s water-transport network. We further evaluated variation in the *D*-*L* scaling relationship among sites and individuals. By verifying the tip-to-base widening pattern in this “living fossil” gymnosperm, our findings clarify how *Ginkgo biloba* maintains hydraulic efficiency despite lacking vein anastomoses. This study provides empirical evidence for the structural optimization of tracheid-based hydraulic systems and establishes a baseline for comparing hydraulic strategies between dichotomous and reticulate venation architectures. The results also advance our understanding of leaf-level water supply optimization from petiole to distal vein terminals.

## 2. Materials and Methods

### 2.1. Sample Collection

**Figure 1:**
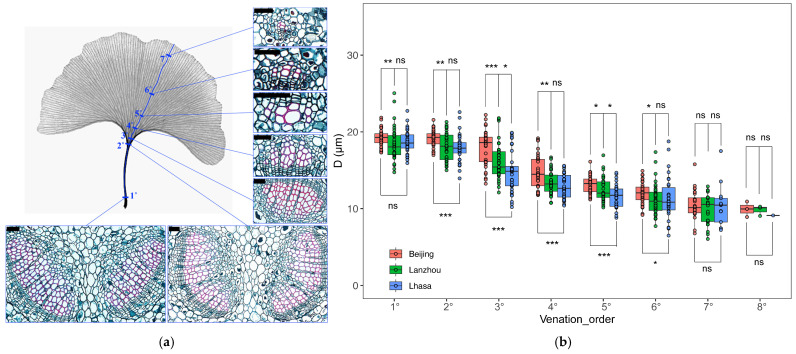
(**a**) Illustrates the sampling scheme for different vein orders and the anatomical structure of tracheids in each order. Veins are clearly labeled in blue in the figure, and the blue numbers represent vein order: 1° represents the primary vein, sampled at the base of the petiole. 2° denotes the secondary vein at the junction between the leaf blade and petiole, where two distinct veins are clearly visible. 3° refers to the tertiary vein formed by the bifurcation of a secondary vein. This hierarchical branching pattern continues until no further subdivision is possible. Bar (top left corner of each micrograph), 50 μm. (**b**) Illustrates the tracheid hydraulic diameter (*D*) across different vein orders at the three sampling sites. Significant differences among sampling sites were determined by one-way ANOVA. Asterisks indicate significant differences between the two sampling sites. ***, *p* < 0.001; **, *p* < 0.01; *, *p* < 0.05; ns, not significant.

From 1 to 7 July 2025, *Ginkgo biloba* healthy individuals with normal growth, no obvious disease or mechanical damage, and similar canopy growth status were randomly selected at each site, avoiding edge trees with extreme microenvironmental interference; tree characteristics including diameter at breast height (DBH) were recorded in Table 1. Six fully expanded, mature and healthy leaves without disease spots, insect pests, or mechanical damage were selected from the outer canopy; leaves were at the same developmental stage with dark green color and intact shape. Leaves were immediately fixed in FAA fixative (formalin-70% ethanol-acetic acid mixture at a volume ratio of 5:90:5, Wuhan Servicebio Technology Co., Ltd, Wuhan, China) for more than 24 h. Subsequently, 0.5 cm tissue segments were cut at each blue bar marked in Figure 1, with their growth directions labeled. Vein order classification was performed following the standardized developmental-based method proposed by Sack, et al. [19], with primary veins (1°) defined as the main vein at the petiole base, and each subsequent higher vein order representing a successive dichotomous branch from the lower order, until terminal minor veins were identified. Specifically, one vein was randomly selected on each leaf; along this vein from the leaf base to the leaf tip, one point was randomly chosen on each vein order to measure tracheid diameter. Each vein contains several tracheids, and all measurable tracheids in the selected tissue segment were measured for statistical analysis. Meanwhile, the distance from each sampling point to the corresponding leaf tip was accurately measured.

### 2.2. Paraffin Section Preparation and Tracheid Diameter Measurement

The cut leaf tissues were dehydrated sequentially in 70%, 85%, 90%, and 100% ethanol (Sinopharm Chemical Reagent Co., Ltd., Shanghai, China) for 2 h each. Thereafter, the leaf tissues were cleared by immersion in a 1:1 mixture of 100% ethanol and xylene (Sinopharm Chemical Reagent Co., Ltd., Shanghai, China), followed by pure xylene, for 2 h each. The tissues were then embedded in paraffin with a melting point of 52–54 °C, sectioned at a thickness of 5 μm using a sliding microtome (Leica SM 2000R, Leica Inc., Wetzler, Germany), and stained with Safranin (Absin Bioscience Inc., Shanghai, China)-Alcian Blue (ScyTek Laboratories Inc., Logan, UT, USA) to prepare permanent sections. The leaf tissue sections were scanned using a Pannoramic slide scanner (Pannoramic 250 Flash III DX, 3DHISTECH Ltd., Budapest, Hungary), and images were analyzed using the 3DHISTECH CaseViewer software (Version 2.4). All tracheids in each leaf tissue sample were measured, and the average value was calculated.

### 2.3. Data Analysis

All statistical analyses were performed using R software (version 4.5.2; R Core Team, 2025) [22]. The allometric relationship between tracheid diameter (*D*) and distance from the tip (*L*) was analyzed via standardized major axis (SMA) regression of log-transformed variables (ln(*D*)~ln(*L*)), using the sma function in the smatr package for R. Standardized Major Axis (SMA) regression was selected instead of Ordinary Least Squares (OLS) regression because SMA regression is suitable for bivariate allometric analysis where both *D* and *L* are random variables with measurement errors, while OLS regression assumes only the dependent variable has error and is prone to biased slope estimation for functional trait scaling relationships. SMA regression is widely recommended in plant hydraulic allometry studies to accurately estimate scaling exponents between functional traits [7,23]. We tested for homogeneity of slopes and Y-intercepts of the fitted SMA lines across different groups (i.e., vein orders, sampling sites, and individual trees). Analyses of Y-intercept homogeneity were only performed when slope homogeneity was confirmed among groups. The ggplot2 package (version 4.0.1) was used for visualizing the allometric relationships. This analysis aimed to explore the axial widening pattern of tracheids from the leaf tip to the base across different vein orders, and to evaluate whether tracheids follow a consistent axial widening principle across individual trees and sampling sites.

## 3. Results

### 3.1. Tracheid Diameter (D) Variation Among Vein Orders

The collected *Ginkgo biloba* leaves had up to eight vein orders. From the petiole to the leaf tip, tracheid diameter (*D*) gradually decreased with increasing vein order. For all vein orders except 7° and 8°, significant differences in *D* were detected among the three sampling sites, with an overall trend of *D* being Beijing > Lanzhou > Lhasa (Figure 1).

### 3.2. Allometric (ln-ln) Relationships Between Tracheid Diameter (D) and Hydraulic Path Length (L)

**Figure 2:**
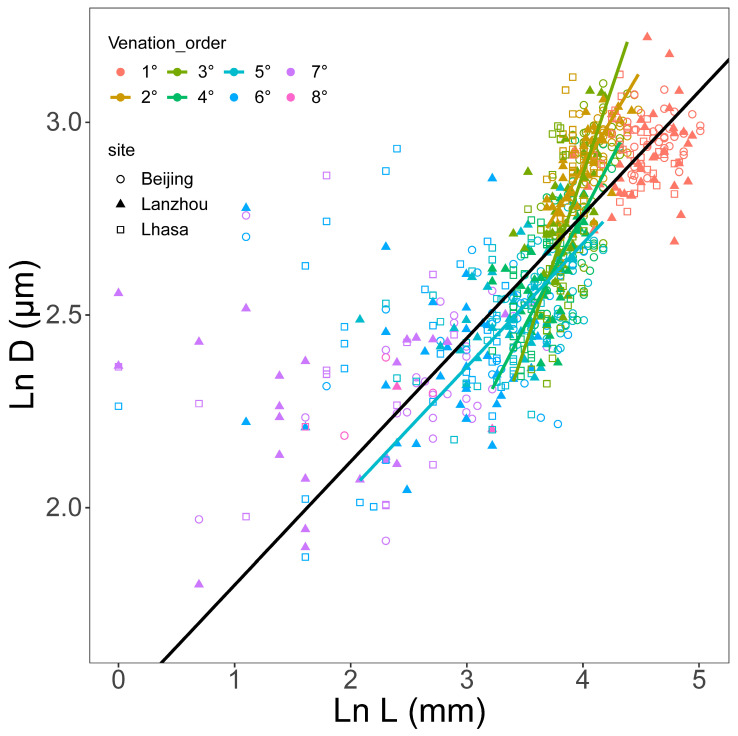
Allometric (ln-ln) relationships between tracheid hydraulic diameter (*D*) and hydraulic path length (*L*) across vein orders, based on 108 *Ginkgo biloba* leaves. The black line shows the overall regression. Colored lines represent different vein orders. Only lines with significant regression relationships (*p* < 0.05) are shown (see Table 2 for details).

**Figure 3:**
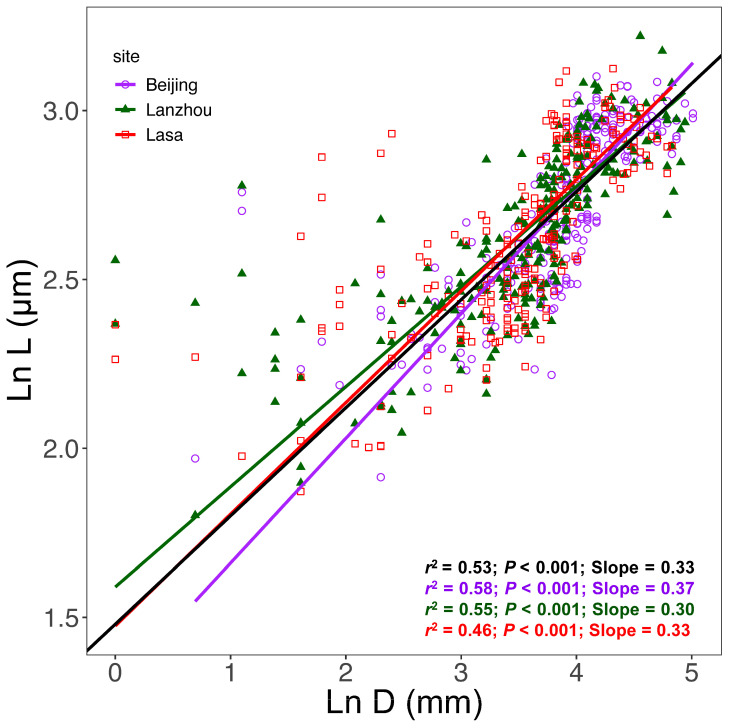
Allometric (ln-ln) relationship between tracheid hydraulic diameter (*D*) and hydraulic path length (*L*) for each sampling site, based on 108 *Ginkgo biloba* leaves. The black line represents the overall regression, and colored lines denote different sampling sites (see Table 2 for details).

**Figure 4:**
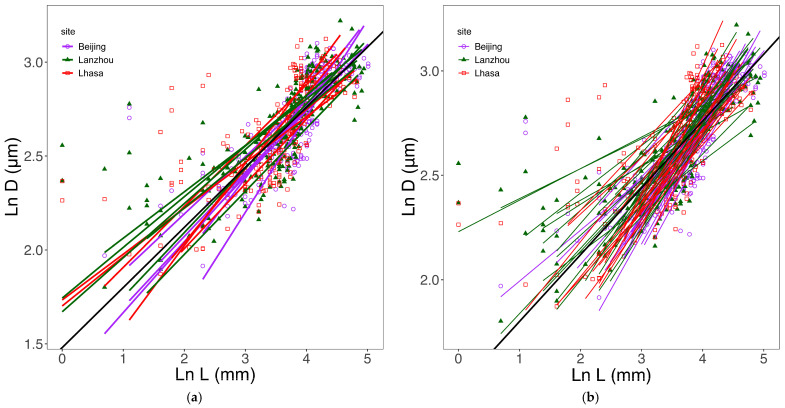
Allometric (ln-ln) relationships between tracheid hydraulic diameter (*D*) and hydraulic path length (*L*) for each of the 18 trees (**a**) and each of the 108 leaves (**b**). The black line represents the overall regression. Each colored line corresponds to one tree or one leaf (see Table 2 for details).

Across all 108 leaves, tracheid diameter (*D*) showed a significant allometric scaling relationship with hydraulic path length (*L*) (*r*^2^ = 0.53, *p* < 0.001) (Figure 2, Figure 3 and Figure 4, Table 2). Although the overall allometric trend was conserved across different grouping categories, SMA regression slopes varied significantly among vein orders (Figure 2), sampling sites (Figure 3), individual trees, and leaves (Figure 4).

When classified by vein order, *D* scaled significantly with *L* in 2°, 3°, 4°, and 5° veins, but no significant relationship was detected in 1°, 6°, 7°, and 8° veins (*p* < 0.05) (Figure 2, Table 2).

Among the three sampling sites, *D* was significantly and positively correlated with *L* within each site, and the scaling exponents differed significantly among sites (*p* < 0.001) (Figure 3, Table 2).

*D* scaled significantly with *L* in all 18 individual trees (*p* < 0.001) and in most of the 108 leaves (*p* < 0.05) (Figure 4, Table 2 and Table S1).

## 4. Discussion

The observed tip-to-base widening of tracheids in *Ginkgo biloba* leaves provides a mechanistic solution to the inherent hydraulic constraints of long-distance water transport. As dictated by the Hagen-Poiseuille law, hydraulic conductance is proportional to the fourth power of the conduit diameter (*D*). This geometric relationship implies that even incremental axial widening significantly offsets the cumulative friction and pressure drop encountered as water moves toward the leaf apex [10,11]. This structural optimization ensures that the leaf margin, despite being the furthest from the water source, receives sufficient hydration to maintain stomatal conductance and photosynthetic rates [24].

A key finding of this study is the non-significant scaling relationship observed in the 1°, 6°, 7°, and 8° vein orders, which contrasts with the robust allometry found in 2–5° veins. Physiologically, this divergence reflects functional specialization among different hierarchical levels. The 1° vein (petiole) serves as the primary structural support; consequently, its tracheid diameter is likely constrained by mechanical requirements to support the leaf lamina against wind and gravity, rather than being strictly optimized for tip-to-base hydraulic scaling [25]. Conversely, the lack of significant scaling in the highest vein orders (6–8°) suggests a shift in hydraulic priority at the terminal leaf margins. At these distal scales, water transport transitions from longitudinal bulk flow to lateral distribution into the mesophyll. The minimal variation in hydraulic path length (*L*) within these fine veins reduces the selective pressure for axial widening. Furthermore, tracheid dimensions in terminal veins may be spatially restricted by the thinning lamina at the leaf margin and the need to maintain high hydraulic safety to prevent embolism at the air-water interface, where tension is highest [24,26]. In contrast, the 2–5° veins represent the primary hydraulic pathways of the leaf, where strict coordination between *D* and *L* is essential to minimize the total hydraulic resistance of the expanding leaf lamina.

Unlike the interconnected reticulate venation of most angiosperms, such as *Populus* or *Acer* species, the open dichotomous system of *Ginkgo biloba* lacks hydraulic redundancy. In reticulate networks, abundant cross-links (anastomoses) allow for bypass flow if a primary conduit is compromised by embolism [24,27]. However, in the non-anastomosing architecture of *Ginkgo biloba*, the hydraulic pathways are essentially isolated within each vein order. In the absence of redundant flow paths, the observed *D*–*L* scaling relationship represents an indispensable evolutionary adaptation to minimize longitudinal resistance along isolated hydraulic routes in a system where transport bottlenecks cannot be circumvented [26,28].

Furthermore, the decrease in tracheid diameter with increasing vein order aligns with Murray’s Law, which suggests that the vascular system is optimized to minimize the combined costs of tissue investment and fluid transport power [29]. In the distal 6–8° veins, the reduced tracheid size likely reflects a trade-off between hydraulic efficiency and the mechanical constraints of the thin laminar margin. By tapering the conduits, *Ginkgo biloba* effectively manages the distribution of water potential across the leaf surface, preventing excessive tension in the terminal tracheids while supporting the structural integrity of the fan-shaped leaf.

The significant variation in tracheid diameter across the three sites (Beijing > Lanzhou > Lhasa) demonstrates high phenotypic plasticity in *Ginkgo biloba*’s hydraulic traits. The reduction in *D* at higher altitudes and lower temperatures (Lhasa) may be an adaptive response to balance transport efficiency with structural safety. Smaller tracheids are less prone to freeze–thaw-induced embolism [4,12], a critical factor for survival in alpine environments. The divergent *D*–*L* scaling slopes among individual trees further indicate that the rate of conduit widening is not fixed but is modulated by the plant’s immediate microclimate and developmental stage. This stability of the widening pattern at the individual level, despite varying magnitudes, underscores its fundamental role in ensuring consistent water supply across diverse habitats [27,30,31].

Collectively, these results confirm the presence of tip-to-base xylem conduit widening in gymnosperms with dichotomous venation, expanding the phylogenetic generality of this adaptive strategy. This work provides a standardized framework for quantifying *D*-*L* allometry across vein orders, applicable to diverse venation types. While our results confirm the axial widening theory in a primitive gymnosperm, several limitations remain. We focused solely on diameter; however, other traits such as pit density and tracheid wall thickness also significantly influence hydraulic safety and efficiency. Future research should examine the ontogenetic development of these conduits to clarify whether the scaling patterns emerge during leaf expansion or are pre-programmed. Expanding the sampling to broader environmental gradients would further validate the generality of our findings regarding intraspecific plasticity.

## 5. Conclusions

This study confirmed that *Ginkgo biloba* leaves exhibit a clear tip-to-base tracheid widening pattern across vein orders, which is consistent with the general hydraulic design of vascular plants. Tracheid diameter (*D*) scaled positively with hydraulic path length (*L*) and decreased with increasing vein order, whereas the allometric slopes differed among sampling sites and individual trees. These findings highlight the generality of the axial widening theory in gymnosperms with dichotomous venation and establish a useful framework for understanding the evolution of leaf venation and hydraulic transport networks.

## Figures and Tables

**Table 1:** Geographical and climatic information of the 3 sampling sites.

Sites	No. Tree	DBH (cm)	Altitude (m)	Longitude (°E)	Latitude (°N)	MAP (mm)	MAT (°C)
Beijing (sampled on 1 July 2025)	1	15	40	116°23′44″	40°04′04″	532.1	13.1
	2	17					
	3	11					
	4	15					
	5	19					
	6	18					
Lanzhou (sampled on 6 July 2025)	1 *	60	1251	103°51′35″	36°02′42″	309	10.9
	2 *	50					
	3	30					
	4	28					
	5	20					
	6	20					
Lhasa (sampled on 7 July 2025)	1	15	3650	91°10′50″	29°38′33″	439.1	9.3
	2	17					
	3	15					
	4	13					
	5	14					
	6	14					

Note. Data were obtained from the China Meteorological Data Service Center [20] and the Dataset of China’s Surface Climate Normals (1991–2020) [21]. DBH: stem diameter at breast height, MAP: mean annual precipitation, MAT: mean annual temperature. * ancient trees over 100 years old, the exact year is unknown.

**Table 2:** Allometric (Ln–Ln) relationships between tracheid hydraulic diameter (*D*) and hydraulic path length (*L*). Intercepts, slopes, 95% confidence intervals (95% CI), *r*^2^ and *p*-values are presented. *p*-values are shown for slope heterogeneity tests in standardized major axis (SMA) regressions.

Ln (*D*)~Ln (*L*)		Intercept (95% CI)	Slope (95% CI)	*r*^2^ (*p*)	Slope Heterogeneity (*p* Value)	Figures
Entire dataset		1.48 (1.42, 1.54)	0.33 (0.31, 0.34)	0.53 ***	--	Figure 2, Figure 3 and Figure 4
Venation orders	1°	1.24 (0.92, 1.56)	0.37 (0.31, 0.45)	0.03 ns	<0.001	Figure 2
	2°	0.86 (0.53, 1.19)	0.51 (0.43, 0.59)	0.31 ***		
	3°	−0.73 (−1.29, −0.16)	0.90 (0.77, 1.05)	0.31 ***		
	4°	0.44 (0.09, 0.78)	0.58 (0.50, 0.68)	0.32 ***		
	5°	1.41 (1.21, 1.60)	0.32 (0.27, 0.38)	0.18 ***		
	6°	1.67 (1.52, 1.83)	0.26 (0.21, 0.31)	0.03 ns		
	7°	1.80 (1.66, 1.94)	0.23 (0.18, 0.29)	0.04 ns		
	8°	2.72 (1.90, 3.54)	−0.18 (−0.68, 0.05)	0.02 ns		
Sites	Beijing	1.29 (1.17, 1.41)	0.37 (0.34, 0.40)	0.58 ***	0.001	Figure 3
	Lanzhou	1.59 (1.50, 1.68)	0.30 (0.27, 0.32)	0.55 ***		
	Lhasa	1.47 (1.35, 1.58)	0.33 (0.30, 0.37)	0.46 ***		
No. Tree	BJ1	1.39 (1.17, 1.61)	0.34 (0.29, 0.40)	0.73 ***	<0.001	Figure 4
	BJ2	1.58 (1.30, 1.87)	0.30 (0.24, 0.39)	0.42 ***		
	BJ3	1.35 (0.99, 1.70)	0.35 (0.27, 0.45)	0.37 ***		
	BJ4	1.30 (1.07, 1.53)	0.37 (0.31, 0.43)	0.75 ***		
	BJ5	0.67 (0.35, 0.98)	0.51 (0.44, 0.59)	0.78 ***		
	BJ6	1.11 (0.79, 1.44)	0.42 (0.35, 0.51)	0.62 ***		
	LZ1	1.41 (1.14, 1.68)	0.34 (0.28, 0.42)	0.58 ***		
	LZ2	1.82 (1.57, 2.06)	0.25 (0.19, 0.32)	0.39 ***		
	LZ3	1.30 (1.08, 1.53)	0.34 (0.28, 0.40)	0.68 ***		
	LZ4	1.67 (1.47, 1.87)	0.29 (0.23, 0.35)	0.58 ***		
	LZ5	1.69 (1.53, 1.85)	0.27 (0.22, 0.31)	0.75 ***		
	LZ6	1.74 (1.52, 1.97)	0.27 (0.22, 0.33)	0.59 ***		
	LS1	1.58 (1.32, 1.85)	0.32 (0.26, 0.41)	0.51 ***		
	LS2	1.25 (0.87, 1.63)	0.39 (0.30, 0.51)	0.36 ***		
	LS3	1.14 (0.88, 1.41)	0.44 (0.37, 0.52)	0.73 ***		
	LS4	1.70 (1.45, 1.95)	0.26 (0.20, 0.34)	0.40 ***		
	LS5	1.73 (1.50, 1.97)	0.24 (0.19, 0.31)	0.53 ***		
	LS6	1.32 (1.03, 1.60)	0.34 (0.27, 0.43)	0.60 ***		

Note. CI, confidence intervals; ***, *p* < 0.001 (e.g., the slope was significantly different from one another); ns, not significant; --, not detected.

## Data Availability

Raw data will be made available as Supplementary Materials upon request.

## Supplementary Materials

The following supporting information can be downloaded at: https://www.mdpi.com/article/10.3390/biology15080598/s1, Table S1: Allometric (Ln–Ln) relationships between tracheid hydraulic diameter (*D*) and hydraulic conductance path length (*L*) per leaf.

## Abbreviations

The following abbreviations are used in this manuscript:

*D*	Tracheid hydraulic diameter
*L*	Hydraulic conductance path length
