# Supplementary material for: Axial Tracheids Widening Across Vein Orders in Ginkgo biloba Leaves and Their Relationship with Hydraulic Path Length
Source: Biology (Basel). 2026 Apr 10;15(8):598. doi: 10.3390/biology15080598 (PMC13113127; doi:10.3390/biology15080598)
Supplement: Supplementary file 1 [file biology-15-00598-s001.zip › biology-4205822-supplementary.pdf]

Table S1. Allometric (ln - ln) relationships between tracheid hydraulic diameter (D) and hydraulic path length (L) per leaf.

No.Leaf	intercept	intercept_lower	intercept_upper	slope	slope_lower	slope_upper	r^2	*P* value
BJ1_1	1.40	0.78	2.02	0.34	0.21	0.53	0.77	0.004
BJ1_2	0.98	0.13	1.83	0.45	0.28	0.73	0.81	0.006
BJ1_3	1.26	0.17	2.34	0.37	0.18	0.74	0.70	0.037
BJ1_4	1.61	0.94	2.27	0.30	0.17	0.53	0.72	0.016
BJ1_5	1.31	0.45	2.18	0.36	0.20	0.64	0.71	0.018
BJ1_6	1.52	0.94	2.10	0.30	0.19	0.49	0.82	0.005
BJ2_1	2.46	2.07	2.86	0.10	0.04	0.25	0.46	0.139
BJ2_2	1.33	0.50	2.16	0.38	0.22	0.67	0.73	0.014
BJ2_3	1.64	0.78	2.50	0.29	0.13	0.61	0.64	0.056
BJ2_4	0.96	0.42	1.49	0.44	0.33	0.60	0.93	0.000
BJ2_5	1.73	0.80	2.67	0.25	0.10	0.61	0.47	0.132
BJ2_6	0.39	-0.87	1.66	0.63	0.37	1.09	0.75	0.012
BJ3_1	0.46	-0.85	1.77	0.54	0.31	0.94	0.74	0.013
BJ3_2	1.30	0.20	2.41	0.36	0.18	0.73	0.55	0.055
BJ3_3	2.14	1.38	2.89	0.17	0.07	0.45	0.07	0.561
BJ3_4	1.01	-0.09	2.11	0.43	0.24	0.79	0.69	0.020
BJ3_5	1.08	0.03	2.13	0.40	0.22	0.75	0.67	0.024
BJ3_6	0.94	-0.41	2.30	0.46	0.23	0.92	0.71	0.036
BJ4_1	0.96	-0.22	2.13	0.44	0.23	0.85	0.64	0.032
BJ4_2	0.55	-0.35	1.45	0.57	0.38	0.86	0.87	0.002
BJ4_3	1.75	1.50	2.00	0.24	0.19	0.32	0.94	0.000
BJ4_4	0.61	-0.73	1.96	0.53	0.29	0.96	0.79	0.018
BJ4_5	0.85	-0.39	2.08	0.49	0.27	0.90	0.69	0.020
BJ4_6	1.28	1.08	1.48	0.38	0.33	0.43	0.98	0.000
BJ5_1	0.98	0.27	1.68	0.44	0.30	0.65	0.84	0.001
BJ5_2	0.65	-0.18	1.49	0.52	0.35	0.77	0.91	0.003
BJ5_3	0.79	-0.79	2.38	0.47	0.22	1.00	0.49	0.082
BJ5_4	0.36	-1.19	1.92	0.58	0.31	1.08	0.77	0.021
BJ5_5	0.52	-0.51	1.55	0.54	0.35	0.84	0.84	0.004
BJ5_6	0.46	-0.69	1.62	0.57	0.36	0.90	0.83	0.005
BJ6_1	0.88	-0.06	1.81	0.48	0.29	0.78	0.80	0.006
BJ6_2	1.61	0.92	2.29	0.29	0.16	0.52	0.63	0.019
BJ6_3	0.65	-1.28	2.58	0.55	0.25	1.22	0.60	0.070
BJ6_4	1.60	1.12	2.08	0.33	0.22	0.49	0.88	0.002
BJ6_5	0.79	-0.23	1.81	0.50	0.30	0.83	0.79	0.008
BJ6_6	0.60	-0.68	1.88	0.53	0.29	0.94	0.71	0.017
LZ1_1	1.42	0.52	2.32	0.34	0.19	0.63	0.68	0.023
LZ1_2	1.17	0.04	2.29	0.41	0.21	0.78	0.63	0.032
LZ1_3	1.19	0.49	1.88	0.41	0.26	0.63	0.85	0.003
LZ1_4	1.41	0.81	2.02	0.33	0.21	0.52	0.78	0.004
LZ1_5	0.74	-0.11	1.60	0.50	0.33	0.77	0.90	0.004
LZ1_6	1.78	1.01	2.55	0.25	0.12	0.53	0.34	0.132
LZ2_1	1.31	0.69	1.94	0.36	0.22	0.57	0.88	0.006
LZ2_2	1.68	0.94	2.42	0.28	0.14	0.55	0.72	0.033
LZ2_3	1.35	0.55	2.15	0.37	0.22	0.63	0.84	0.010
LZ2_4	2.17	1.36	2.98	0.17	0.06	0.51	0.07	0.615
LZ2_5	2.24	1.85	2.64	0.14	0.07	0.29	0.58	0.048
LZ2_6	0.68	-0.66	2.02	0.52	0.28	0.95	0.78	0.020

LZ3_1	1.28	0.81	1.74	0.36	0.25	0.52	0.89	0.001
LZ3_2	0.96	-0.05	1.97	0.43	0.23	0.79	0.68	0.022
LZ3_3	1.40	0.85	1.95	0.30	0.19	0.49	0.81	0.006
LZ3_4	0.85	-0.03	1.72	0.46	0.28	0.76	0.86	0.008
LZ3_5	1.69	1.09	2.29	0.22	0.11	0.44	0.58	0.045
LZ3_6	0.98	-0.08	2.03	0.43	0.23	0.81	0.66	0.026
LZ4_1	0.95	0.20	1.71	0.47	0.30	0.73	0.84	0.004
LZ4_2	1.52	1.18	1.86	0.31	0.23	0.43	0.92	0.001
LZ4_3	1.31	0.84	1.78	0.37	0.26	0.53	0.90	0.001
LZ4_4	2.18	1.70	2.66	0.15	0.06	0.37	0.25	0.249
LZ4_5	1.82	1.46	2.18	0.26	0.17	0.39	0.86	0.003
LZ4_6	1.60	1.01	2.19	0.34	0.20	0.58	0.76	0.011
LZ5_1	2.05	1.72	2.39	0.16	0.09	0.28	0.74	0.013
LZ5_2	1.23	0.84	1.61	0.40	0.31	0.52	0.96	0.001
LZ5_3	0.85	0.35	1.34	0.48	0.37	0.63	0.98	0.001
LZ5_4	1.68	1.18	2.19	0.26	0.16	0.45	0.84	0.010
LZ5_5	1.99	1.68	2.29	0.19	0.12	0.29	0.83	0.004
LZ5_6	1.52	0.96	2.07	0.31	0.19	0.50	0.81	0.006
LZ6_1	1.65	1.14	2.16	0.29	0.19	0.46	0.85	0.003
LZ6_2	2.23	1.78	2.68	0.15	0.07	0.31	0.67	0.046
LZ6_3	1.96	1.56	2.36	0.23	0.14	0.37	0.87	0.007
LZ6_4	0.99	0.45	1.54	0.48	0.35	0.65	0.93	0.001
LZ6_5	1.31	0.05	2.57	0.35	0.15	0.80	0.55	0.089
LZ6_6	0.52	-1.39	2.43	0.57	0.26	1.22	0.63	0.059
LS1_1	1.06	-0.38	2.49	0.45	0.21	0.98	0.45	0.102
LS1_2	1.70	1.34	2.07	0.31	0.22	0.44	0.91	0.001
LS1_3	0.31	-0.25	0.87	0.65	0.51	0.82	0.98	0.001
LS1_4	1.57	0.55	2.58	0.33	0.15	0.73	0.59	0.076
LS1_5	1.99	1.30	2.67	0.23	0.10	0.51	0.39	0.135
LS1_6	1.32	0.72	1.92	0.38	0.25	0.59	0.85	0.003
LS2_1	1.83	0.76	2.90	0.25	0.10	0.68	0.02	0.768
LS2_2	1.23	0.83	1.63	0.37	0.27	0.50	0.95	0.001
LS2_3	0.80	-0.01	1.61	0.54	0.35	0.82	0.90	0.004
LS2_4	1.04	0.14	1.95	0.43	0.25	0.73	0.76	0.010
LS2_5	0.88	0.23	1.54	0.47	0.32	0.68	0.89	0.001
LS2_6	1.83	0.47	3.19	0.28	0.09	0.85	0.06	0.635
LS3_1	0.79	0.14	1.43	0.52	0.37	0.73	0.91	0.001
LS3_2	0.41	-0.73	1.56	0.65	0.41	1.04	0.82	0.005
LS3_3	1.45	1.03	1.88	0.36	0.26	0.51	0.91	0.001
LS3_4	0.75	-0.38	1.88	0.55	0.32	0.95	0.75	0.011
LS3_5	1.77	1.36	2.19	0.27	0.18	0.42	0.85	0.003
LS3_6	0.26	-1.94	2.45	0.64	0.28	1.43	0.58	0.079
LS4_1	1.31	0.88	1.74	0.35	0.25	0.50	0.94	0.002
LS4_2	1.95	0.92	2.97	0.22	0.07	0.65	0.10	0.551
LS4_3	1.86	0.79	2.93	0.22	0.07	0.65	0.10	0.537
LS4_4	2.10	1.60	2.59	0.17	0.07	0.37	0.39	0.134
LS4_5	1.36	0.62	2.11	0.32	0.17	0.59	0.79	0.019
LS4_6	1.15	0.28	2.02	0.41	0.23	0.70	0.83	0.012
LS5_1	1.40	0.52	2.28	0.33	0.17	0.62	0.77	0.023
LS5_2	0.40	-0.54	1.34	0.58	0.38	0.87	0.91	0.003
LS5_3	0.98	-1.11	3.07	0.44	0.16	1.22	0.84	0.083
LS5_4	1.63	0.17	3.09	0.28	0.09	0.88	0.40	0.253

LS5_5	1.45	0.19	2.70	0.32	0.13	0.81	0.66	0.096
LS5_6	2.10	1.63	2.56	0.15	0.07	0.32	0.65	0.054
LS6_1	1.23	0.61	1.85	0.36	0.23	0.58	0.88	0.006
LS6_2	1.09	0.30	1.89	0.39	0.23	0.66	0.85	0.009
LS6_3	1.01	-1.35	3.36	0.44	0.13	1.42	0.35	0.293
LS6_4	1.61	0.59	2.63	0.28	0.11	0.68	0.46	0.138
LS6_5	1.35	-0.03	2.72	0.32	0.11	0.87	0.58	0.137
LS6_6	1.52	0.54	2.51	0.30	0.13	0.69	0.55	0.093
